# Does Combining Antenatal Care Visits at Health Posts and Health Centers Improve Antenatal Care Quality in Rural Ethiopia?

**DOI:** 10.4314/ejhs.v33i1.5S

**Published:** 2023-04

**Authors:** Habtamu Kebebe Kasaye, Tegene Legese Dadi, Mekdes Tigistu Yilma, Mulusew G Jebena, Girmay Medhin, Getnet Mitike Kassie, Frehiwot Bekele, Frehiwot Nigatu, Alula M Teklu

**Affiliations:** 1 Department of Midwifery, Institute of Health Sciences, Wallaga University, Ethiopia; 2 School of Public Health, Hawassa University, Ethiopia; 3 MERQ Consultancy PLC, Addis Ababa, Ethiopia; 4 Department of Public Health, Institute of Health Sciences, Wallaga University, Ethiopia; 5 Department of Epidemiology, Institute of Health, Jimma University; 6 Aklilu Lemma Institute of Pathobiology, Addis Ababa University, Ethiopia; 7 International Institute for Primary Health Care- Ethiopia (IIfPHC-E); 8 Ethiopian Public Health Institute (EPHI), Addis Ababa, Ethiopia

**Keywords:** quality of health care, antenatal care, primary health care

## Abstract

**Background:**

Even though quality maternal care is crucial for the well-being of women and their newborns, the inferior quality of antenatal care in rural Ethiopia is a timely concern. This study aimed to investigate the effects of combining antenatal care visits at health posts and health centers on improving antenatal care quality in rural Ethiopia.

**Methods:**

Using the 2019 Ethiopia Health Extension Program assessment done by MERQ, we extracted and analyzed the survey responses of 2,660 women who had received at least one antenatal visit from a primary health care unit. We measured the cumulative count of quality of antenatal care using the Donabedian model. To model the differences in the quality of antenatal care at health posts and health centers, we used zero-truncated Poisson regression and reported incidence risk ratios with their 95% confidence intervals.

**Results:**

The quality of antenatal care increased by 20% (adjusted IRR= 1.20 [1.12–1.28]) when antenatal care reception was mixed at health posts and health centers, compared to those who received all antenatal care only from health posts. Quality differences based on socioeconomic status and setting variations were observed as predictors of quality of care, even if women received antenatal care at both health posts and health centers.

**Conclusions:**

Combining antenatal care provision from health posts and health centers should be sustained as one of the antenatal care quality improvement strategies in rural parts of Ethiopia while ensuring the equitable provision of quality care across socioeconomic groups and between agrarian and pastoral settings.

## Introduction

Access to quality essential health care services is key to universal health coverage (1), which is a target for Sustainable Development Goal (SDG) 3 (2). The provision of quality antenatal care (ANC) is one of the World Health Organization's (WHO) recommendations to identify, prevent, and manage pregnancy-related complications (3). The five-year Health Sector Transformation Plan of Ethiopia also aims to ensure universal primary health coverage and improve access to quality health services (4).

Ensuring the quality of essential health care services has tremendous benefits. For instance, ANC quality influences women's health and well-being, and that of their babies, during and after pregnancy (5). It also enhances the uptake of health facility services such as skilled birth attendance and increases women's health-seeking behavior (6). Quality ANC could also enable early detection and management of obstetric and non-obstetric complications and thereby minimize maternal mortality (3,7), and it helps to alleviate preventable stillbirths and newborn deaths (8,9).

However, evidence shows that the quality of ANC services is inadequate and inequitable, especially in vulnerable groups in low- and middle-income countries such as Ethiopia (10,11). It varies by poverty level—most impoverished women receive poor quality care, unlike the wealthiest women (11,12). Although ANC coverage has improved in Ethiopia, the quality of ANC is lacking. ANC coverage has been enhanced from 27% in 2000 to 75% in 2019 (13,14), but an analysis of demographic and health survey data by Dinsa *et al.* showed that only one in three women received quality care (15).

Studies have identified several barriers to receiving quality ANC, ranging from social determinants to providers' competencies (10,16,17). Social determinants such as living in a rural area, being categorized as a member of the lowest quantile on the wealth index, and the low educational status of women were among negative predictors of receiving quality ANC (1,10,11,18). Health facilities' readiness in terms of resources and competently skilled providers were identified as enhancing the quality of ANC (16,17). The level of readiness for providing quality ANC in Ethiopia was low across health centers (HCs) and health posts (HPs), where most women access health care, compared to hospitals (19).

The significance of the ANC quality problem is clear at HPs, where the primary care providers are health extension workers (HEWs) who have limited experience and skills (4). To overcome the practical quality problem at HPs, Ethiopia's government introduced combined ANC services at HCs and HPs. However, the extent to which combining HC and HP visits addresses the quality gap in the provision of ANC at HPs is not clearly understood (5,20). To the best of our knowledge, no published evidence shows whether combining ANC at HPs and HCs in rural parts of Ethiopia has effect on ANC quality in these areas. Therefore, this study aimed to investigate the effects of combining ANC visits across HPs and HCs on care quality in rural Ethiopia.

## Materials and Methods

**Study context and setting**: This paper is based on data collected for a nationwide survey that covered rural areas of Ethiopia. During the field survey, the country was administratively divided into nine regions and two city administrations. These regions have been further subdivided into zones, woredas or districts, and kebeles (the lowest tier of government administrative units). Based on the 2007 Ethiopian population and housing census projections, the average projected population of Ethiopia in 2020 was 100.8 million, and about 78% of the population lived in rural areas (21).

Ethiopian health service delivery is structured in a three-tier system of primary, secondary, and tertiary levels. The primary level, or primary health care (PHC) unit, comprises HPs, HCs, and a primary hospital that provides services for about 100,000 people. The Health Extension Program (HEP) is structured, implemented, and managed under PHC units (4). Almost all kebeles in all regions have at least one HP staffed with two salaried HEWs. HEWs complete a one-year training program focused on family health services, disease prevention and control, hygiene and environmental sanitation, health education, and communication (22). The overall health system operates with more than 17,000 HPs, 3,600 HCs, and 300 public hospitals and close to 160,000 health professionals and 40,000 HEWs (23). This study focused on ANC service receipt in PHC units, especially at HPs and HCs, throughout rural areas.

**Data source and study design**: We used data extracted from the 2019 national assessment of the Ethiopian HEP. The HEP assessment survey was a comprehensive nationwide cross-sectional community-based study conducted from March to May 2019 (24).

**Study participants**: We extracted the data related to women with a completed pregnancy from 7,043 households (HHs) and included in the HEP assessment to explore their ANC service uptake. Data from the women's catchment HPs and HCs were collected separately and merged with the HH survey data.

**Sampling and data acquisitions procedures**: The rural HEP assessment covered sixty-two randomly selected woredas, or districts, from nine regions; 343 HPs nested in these woredas (six within each woreda); 179 HCs that supervise the study HPs; and 7,043 HHs (selected from three kebeles within each woreda and 34 HHs in each kebele). The women were the primary respondents for the HH data. In this analysis, we used quantitative data from women who had ANC visits for a recent pregnancy (N = 2,660). The HEP survey used data collection tools adapted from standardized tools from myriad studies, and the data were collected using the Open Data Kit (ODK) software. Participants were asked about their experiences regarding the care they received from health facilities during their most recent pregnancy within the five years preceding the survey.

### Measurements

**Outcome variable**: The quality of ANC participants received was the outcome of interest. This outcome was defined based on the Donabedian model, which is a comprehensive framework that assesses health care quality using input, processes, and the outcomes of care (25). The structure, process, and outcome dimensions were used to compute ANC quality. The quality tracers included in the structure were based on the recommendations of national quality initiatives and taken as foundation/input indicators in *Ethiopian National Healthcare Quality and Safety Strategy*. Thus, the structure component includes the combined effects of health facilities and capacity building actions such as referral, supervision, and monitoring of services (26). These are essential inputs for a high-quality health system as a foundation for the process of the care.

The remaining two categories, process, and outcome were based on WHO recommendations and their desired ANC outcomes (27,28). Components of antenatal care service provision, such as blood pressure measurement, tetanus toxoid vaccination, iron foliate administration, advice on danger signs and complication readiness, and advice on birth readiness, were included in process categories. The proportion of women who reported being aware of danger signs, attending four or more ANC follow-ups, and having childbirth attended by skilled providers in health facilities were included in the desired outcome category. To determine whether the woman was aware of pregnancy danger signs, which is like labor and birth danger signs, a woman who reported at least one danger sign was assumed to be aware of danger signs. Despite the fact that most women are expected to be aware of the majority of danger signs, only a small percentage (32.6%) of women reported being aware of danger signs in this national survey. This indicates that the possibility of positively biasing antenatal care quality is minimal.

As stated in previous paragraphs, based on the Donabedian model, quality of care is not a simple variable that can be measured as a dichotomous (Yes/No) variable because multifaceted indicators influence quality. Therefore, in this study, the overall quality of ANC was measured as a count variable composed of six variables from the structure, ten from the process, and three from the outcome indicators. The indicators were considered to have equal weight on completeness of service delivery and the availability of a specific condition. The measurement resulted in an overall score that ranges from 1 to 19. The internal consistency of these tracers was assessed, and the coefficient alpha was 0.75. The variables (indicators) used to measure the quality of ANC are summarized in Table 1.

**Table 1:** Description of tracers used to measure the quality of antenatal care received by women from primary health care units of rural Ethiopia based on the Donabedian model, 2019

Components of indicators	Indicators used to measure overall quality of ANC provided*
Structure (6 variables)	Availability of ANC-related services at health facilities HCs supervise catchment HPs HCs supply HPs with program drugs Performance review team review the performance of primary health care unit Presence of Level IV HEWs and mix of other health professionals such as nurses and midwives at HPs Referral system of pregnant women
Process (10 variables)	Blood pressure measured Tetanus toxoid vaccination provided Given iron foliate Advised on danger signs and complication readiness Advised on birth preparedness Tested for HIV/AIDS Counseled about nutrition Blood sample taken Urine sample taken Given drugs for intestinal parasites
Outcome (3 variables)	Women attended 4+ ANC visits Women know at least one danger sign during pregnancy Gave birth at health facility

* *Note*. All measured as *yes* or *no*, with yes = 1 and no = 0.

ANC: antenatal care; HC: health center; HP: health post; HEWs: health extension workers

**Primary exposure variable**: The primary explanatory variable was the health facility that the women visited for ANC (place of ANC), as we sought to determine the effect of ANC follow-up at HCs and HPs compared to those who received ANC follow-up only at health posts. It is a factor variable categorized into two: all ANC visits at HPs, and visits at HPs and HCs. Because accessible rural health care is available from HPs and HCs, we did not include higher tier public facilities and private facilities when defining the exposure variable.

**Other independent variables**: Other independent variables were categorized under social determinants, the women's obstetrics characteristics, and structure-related variables. Social determinants include region by livelihood, wealth quintiles, educational status, and marital status. The wealth quintiles were determined using principal component analysis (PCA), as described in the survey report (24). Obstetric characteristics comprised parity, bad obstetric history (BOH) computed from the history of abortion and stillbirth, and the number of ANC visits conducted. We also included the presence of competent providers at health care facilities, based on whether providers held a certificate of competence (COC) and whether respective HCs provided training for HEWs.

**Data analysis**: Descriptive statistical summaries, including frequencies and cross-tabulation, were generated and presented in tables and graphs. The computed quality of care was left truncated at zero, so we could not fit the Poisson regression model and we checked for further appropriate models. In the absence of significant evidence of over-dispersion (having a mean quality of ANC of 12.1, a variance of 12.6, and an over-dispersion parameter alpha = 3.39e-16≈0), truncated Poisson regression (TPR)—rather than zero-truncated negative binomial regression (ZTNB)—was selected to model the data.

To identify the effects of combining ANC visits to HPs and HCs, we used the TPR model as recommended by Cameron and Trivedi (29) while controlling the effects of potential confounding variables. To control the bias related to the complex survey design, we computed linearized standard errors in Stata by specifying the survey's key features (30). Considering the survey design, the final TPR model was fitted for only 1,023 cases who conducted all ANC visits at HPs and who combined HP and HC visits. The post-estimation model fitness was tested using the Wald test. The incidence rate ratios (IRRs) and their corresponding 95% confidence intervals (CIs) were reported. Findings were reported as being statistically significant when p values were less than 0.05. All data analysis was performed using Stata 15 (31).

**Ethics approval**: The Institutional Review Board of the Ethiopian Public Health Institute approved the protocol of the national HEP assessment for its ethical standard. The national HEP assessment was conducted thoroughly by adhering to ethical principles of human research. The study participants were informed about the aim and benefits of the study and their participation being voluntary. Confidentiality of the collected information was assured through coding personal identifiers.

## Results

**Description of study participants**: Among the women identified in a survey of 7,043 HHs, 2,660 reported having at least one ANC follow-up at health facilities for a pregnancy in the last five years. The characteristics of these women are summarized in Table 2.

**Table 2:** Characteristics of women who received antenatal care from health facilities in Ethiopia, 2019 (N = 2,660)

Characteristics	Responses	Unweighted N (%)	Weighted% (95% CI)
Age (years)	< 20	110 (4.1)	2.3 [1.2–4.4]
	20–34	1,841 (69.2)	69.0 [63.6–73.9]
	35–49	709 (26.6)	28.7 [23.6–34.3]
Educational status	No formal education	1,600 (60.1)	56.4 [51.3–61.3]
	Elementary (Grade 1–8)	862 (32.4)	36.1 [31.2–41.2]
	High school and above	198 (7.4)	7.5 [6.2–9.1]
Marital status	Currently married	2,495 (93.8)	95.3 [93.4–96.6]
	Other	165 (6.2)	4.7 [3.4–6.6]
Wealth quintile	Lowest	393 (14.8)	13.8 [10.7–17.6]
	Lower	470 (17.7)	18.1 [14.0–23.1]
	Middle	516 (19.4)	20.3 [17.5–23.4]
	Higher	627 (23.6)	24.4 [20.1–29.3]
	Highest	654 (24.6)	23.3 [18.5–29]
Livelihood	Agrarian	2,028 (76.2)	97.4 [93.8– 98.9]
	Pastoral	632 (23.8)	2.6 [1.1–6.1]

Their ages ranged from 15 to 45 years, and their median age was 29 years (interquartile range of 10 years). Among this group of women, 56% had no formal education, 95% were married, and 2.5% were from pastoral settings.

**Participants' characteristics related to obstetrics and ANC services**: A summary of the characteristics related to participants' pregnancies and ANC service uptake is presented in Table 3.

**Table 3:** Antenatal care attendees by obstetric history and service-related characteristics of women, Ethiopia, 2019 (N = 2660)

Characteristics	Responses	Unweight N (%)	Weighted % [95% CI]
Parity	Nulliparous	69 (2.6)	2.1 [1.3–3.3]
	Multiparous	1,657 (62.3)	63.5 [58.8–67.9]
	Grand-multiparous	934 (35.1)	34.4 [29.7–39.4]
History of stillbirth	Yes	254 (9.5)	9.6 [7.7–11.8]
	No	2,406 (90.4)	90.4 [88.2–92.2]
History of abortion	Yes	379 (14.5)	14.0 [11.9–16.5]
	No	2,229 (85.5)	85.9 [83.5–88.1]
Number of ANC visits (median 4±1)	1	145 (5.4)	4.8 [2.9–7.6]
	2	332 (12.5)	10.2 [8.3–12.4]
	3	791 (29.7)	30.1 [25.2–35.5]
	4+	1,392 (52.3)	54.91 [47.–62.1]
Gestational age (trimester) at first ANC visit	1^st^	1,513 (56.9)	54.2(48.1–60.2]
	2^nd^	971 (36.5)	40.4 [36.4–44.5]
	3^rd^	62 (2.3)	1.9 [1.0–3.4]
	Don't know	114 (4.3)	3.5 [1.5–7.8]
Health facility where ANC received (N = 2018)	All at HP	605 (30.0)	31.34 [23.6–40.3]
	Mixed HP and HC	418 (20.7)	20.2 [16.3–24.8]
	All at HC	785 (38.9)	39.0 [30.8–48.0]
	Mixed HP and GH/PHCF	50 (2.5)	3.4 [1.5–7.1]
	All at GH	132 (6.5)	5.1 [2.5–10.0]
	All at PHCF	28 (1.4)	0.9 [0.4–1.9]

Note: ANC: antenatal care; HP: health post; HC: health center; GH: general hospital; PHCF: private health care facility.

More than a third (34%) of women were grand-multiparous (five and more childbirths), with a median of four childbirths; 14% reported having had an abortion; and 9.6% had experienced a stillbirth. Forty-seven women had experienced both an abortion and a stillbirth. More than half (55%) attended more than three ANC visits, and the same number started ANC within 16 weeks of pregnancy. One-fifth attended ANC visits at both HPs and HCs.

**Figure 1:**
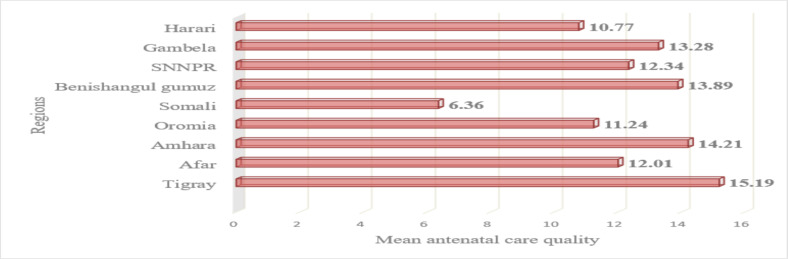
Regional variability in mean antenatal care quality received by pregnant women from health facilities of rural Ethiopia, 2019

**Quality of ANC**: The number of ANC quality components provided to participants ranges from one to 19, with a mean of 12.1 and standard deviation 3.5 at HPs alone and at HPs and HCs combined. ANC quality disparities existed among regions of the country. Women in the Tigray region received the highest quality ANC (15.2), and those in the Somali region received the lowest quality ANC (6.4) as depicted in Figure 1.

The components provided to the women varied depending on where they received ANC. From recommended components of ANC, blood pressure measurement was more likely to be performed for those who visited both types of facilities (X^2^(44) = 23.56, p < 0.001) than those who visited only an HP. Similarly, receiving iron folate (X^2^(44) = 14.02, p<0.001), having a urine sample (X^2^(44) = 14.07, p < 0.001) or blood sample (X^2^(44) = 21.17, p<0.001) tested, and having an HIV test (X^2^ = 49.94, p < 0.001) differed significantly between the two groups (Table 4).

**Table 4:** Components of antenatal care provided to women by health posts and combined health facilities of Ethiopia, 2019 (N = 1023)

Components of care and expected outcome of care	HP only – n (%)	Combined at HP and HC – n (%)	Chi-square (p value)
Blood pressure measured	438 (65.9)	371 (86.0)	23.56 (< 0.001)
Received advice on danger signs	268 (47.1)	221 (47.4)	0.002 (0.961)
Received TT vaccination	461 (79.8)	322 (80.8)	0.111 (0.741)
Received iron foliate	331 (53.4)	324 (77.2)	14.017 (< 0.001)
Urine test done	232 (37.4)	300 (68.8)	14.076 (< 0.001)
Blood test done	286 (43.4)	328 (73.2)	21.17 (< 0.001)
Received drug for deworming	98 (21.2)	89 (20.2)	0.046 (0.832)
Received nutritional counseling	309 (54.1)	285 (63.4)	1.915 (0.173)
HIV test done	246 (37.6)	285 (64.0)	49.943 (< 0.001)
Advised on birth preparedness plan	406 (72.1)	278 (70.2)	0.316 (0.577)
Women knows danger sign	182 (32.1)	152 (31.5)	0.011 (0.917)
Gave birth at health facility	342 (57.9)	272 (66.1)	1.714 (0.197)

*Note*. HP: health post; HC: health center; TT: tetanus toxoid

**Effect of combining ANC at HPs and HCs on quality of care**: Findings from the TPR analysis that compared the effect of place of service delivery are summarized in Table 5.

**Table 5:** Summary of findings from zero-truncated Poisson regression model with quality of antenatal care received from primary health care units in Ethiopia, 2019

Characteristics	Responses	Average quality score	Adjusted IRR (95% CI)
HF attended for ANC	All visits at HPs	10.69	Reference
	Visits mixed – HPs and HCs	13.94	1.20 [1.12–1.28]
Wealth quintile	Lowest	11.54	0.89 [0.81–0.97]
	Lower	12.10	0.93 [0.87–1.03]
	Middle	12.31	0.93 [0.85–1.03]
	Higher	11.74	0.93 [0.86–1.01]
	Highest	12.31	Reference
Age (years)	<20	10.97	Reference
	20–34	11.89	1.11 [0.94–1.31]
	35–49	12.45	1.16 [0.95–1.41]
Educational status	No formal education	11.65	Reference
	Elementary (Grade 1–8)	12.61	1.05 [0.99–1.12]
	Secondary and above	13.19	1.12 [0.99–1.26]
Marital status	Currently married	12.00	Reference
	Other	12.23	0.99 [0.92–1.20]
Parity	Nulliparous	9.33	Reference
	Multiparous	12.23	1.08 [0.84–1.41]
	Grand-multiparous	11.80	1.04 [0.77–1.41]
Livelihood	Agrarian	12.74	Reference
	Pastoral	10.36	0.86 [0.73–0.99]
Bad obstetric history	Yes	12.45	1.00 [0.96–1.05]
	No	11.91	Reference
COC-certified providers	Yes	12.26	1.02 [0.93–1.12]
	No	10.57	Reference
Training of HEWs	Yes	12.12	1.05 [0.98–1.13]
	No	11.95	Reference
Number of ANC visits	1	7.60	Reference
	2	9.64	1.00 [0.72–1.38]
	3	11.06	1.11 [0.86–1.44]
	4+	13.30	1.21 [0.92–1.61]

Note. ANC: antenatal care; HP: health post; HC: health center; HEWs: health extension workers; IRR: incidence rate ratio; COC: certificate of competency

Combined attendance at HPs and HCs increased the ANC provision quality score by 20% (adjusted IRR = 1.20, 95% CI [1.12–1.28]) compared to those who received all ANC from HPs, after adjusting for potential confounding variables. From another view, the quality of ANC varied between the women's wealth quintile and livelihood (agrarian vs. pastoral). For women from the lowest wealth quintile, ANC quality decreased by 11% (adjusted IRR = 0.89, 95% CI [0.81– 0.97]) compared to women from the highest wealth quintile. For women from pastoral areas, ANC quality was 14% lower (adjusted IRR = 0.86, 95% CI [0.73–0.99]) than for women residing in agrarian areas.

## Discussion

In this study, nearly a third of ANC quality indicators were not provided for the women who accessed ANC either at HPs alone or at HPs and HCs. Combining ANC at HPs and HCs increased ANC quality by 20% compared to ANC received only from HPs. We identified inequality in service provision—the quality of ANC varied by wealth quintile and livelihood.

Our findings highlight the gap between improvements in ANC coverage over time and ANC quality. This compromised quality of ANC could be a reason contributing to high home birth in Ethiopia, which is estimated to be more than a half all birth in the country (14). The gap in structural readiness arising from resource scarcity could be the pivotal cause of compromised care quality. To gain benefits from the improvement of ANC quality, such as increased institutional deliveries (6) and reduced stillbirths and neonatal mortality (8,9), designing alternative service requirements is crucial for contexts in which resources are scarce. Equipping health facilities with essential resources to provide the required ANC components, enhancing the desired outcomes, we examined in this study, and improving ANC quality requires stakeholders' attention.

We found that adherence to the Ministry of Health's recommendation to receive ANC from HPs and HCs combined improved the quality of care. The increment in number of quality components of ANC when combining care at facilities might have relatively enhanced supply and resources readiness, including the availability of more skilled providers at HCs (4,19). Although attending ANC visits at facilities with better infrastructure might not guarantee the quality of care (32), and improved quality of ANC derived from visiting two types of facilities could be related to access to enhanced structural readiness. The unfinished agenda of improving the competency of health providers at HPs, that is, of HEWs (4), could also play a role in the relatively lower quality of ANC delivered at HPs. While improving the service quality at HPs is indispensable, the findings of the current study emphasize the need for all regional health bureaus, health facilities, and PHC providers to adhere to the recommendation of the Ministry of Health to combine the provision of ANC at HPs and HCs in areas where providing skilled ANC for all attendant is not possible related to contextual factors existing in rural communities.

We found that wealth quintile independently affected the quality of ANC women received. The poorest women experienced considerably lower mean ANC quality, which was also observed in previous studies (10–12,18). The poorest women might tend not to complete the recommended number of visits reported elsewhere (5) and seek less health care, as evidenced by the health expenditure survey (33). The variability in service provision based on social status motivates one to question service providers' fairness in their work or health facilities' readiness in supplying equitable care through enabling vulnerable groups of service consumers. This is even more concerning when women receive the services for free. One breach of fairness relates to the high incidence of mistreatments, which includes providers prejudices based on economic status, experienced by those seeking health care in the country (34–36). Such unethical behavior of care providers might also cause the poorest women to receive the lower quality of care observed in this study.

We further identified that women in pastoral settings received significantly lower quality ANC than those in agrarian settings. The qualitative findings from Afar and Somali regions show that women from pastoral settings frequently receive care from traditional birth attendants rather than from health care providers (37,38). This tendency and pastoral women's minimal completion of recommended care reduce the probability of them receiving better quality of care. Women's cultural preferences in pastoral areas and their mobile lifestyle might affect their receptiveness to recommended care, which is the core of the quality of care. The way services are being provided in these settings needs to address women's and overall communities' lifestyles and cultural preferences.

One of the current study's strengths is that we assessed the quality of ANC from comprehensive perspectives, including input, content (process), and desired outcome in the rural setting. We did not merely measure those components of care as a proportion of availability of care; as far as is reasonable, we combined all aspects of indicators and computed overall ANC quality in rural parts of the country as recommended in Donabedian model (25).

Nevertheless, our study has limitations. First, we used the services women reported accessing as process indicators, which could have been measured better by actual observations of care provision, including providers' perspectives. However, direct observation carries the risk of social desirability bias. Another limitation is the risk of recall bias, as respondents were asked to recall their experiences within the preceding five years. Recall bias arising from women in reporting incidents at health facilities was noted to be moderate, whereas life experiences such as invasive diagnostic procedures and interventions and prolonged drug taking tend to be reported accurately (39). As a result, we believe that the bias introduced by recalling and relying on women's self-reports as a source of information is minimal.

In conclusion, the association between improved ANC quality and receiving ANC from both HPs and HCs suggests that this practice should be maintained. It is critical that this practice be preserved as an ANC quality improvement strategy in rural Ethiopia until our health systems can provide all ANC visits or contacts by skilled care providers per the WHO recommendations. However, combining ANC at HPs and HCs did not improve service quality inequality. Quality-improvement initiatives should prioritize the vulnerable groups of the community like poorest segments of the population and pastoral communities.
